# Can Antinuclear Antibodies Have a Pathogenic Role in Systemic Sclerosis?

**DOI:** 10.3389/fimmu.2022.930970

**Published:** 2022-06-28

**Authors:** Aurélien Chepy, Louisa Bourel, Vincent Koether, David Launay, Sylvain Dubucquoi, Vincent Sobanski

**Affiliations:** ^1^ Univ. Lille, Inserm, CHU Lille, U1286—INFINITE—Institute for Translational Research in Inflammation, Lille, France; ^2^ CHU Lille, Département de Médecine Interne et Immunologie Clinique, Centre de Référence des Maladies Auto-immunes Systémiques Rares du Nord et Nord-Ouest de France (CeRAINO), Lille, France; ^3^ CHU Lille, Institut d’Immunologie, Lille, France; ^4^ Institut Universitaire de France (IUF), Paris, France

**Keywords:** antibodies, systemic sclerosis, biomarkers, antinuclear antibodies (ANA), pathogenic antibody

## Abstract

Systemic sclerosis (SSc) is a connective tissue disease characterized by extensive fibrosis of the skin and internal organs, associated with vasculopathy and autoimmune features. Antinuclear antibodies (ANA) are found in almost all SSc patients and constitute strong diagnosis and prognosis biomarkers. However, it remains unclear whether ANA are simple bystanders or if they can have a role in the pathophysiology of the disease. One might think that the nuclear nature of their targets prevents any accessibility to autoantibodies. Nevertheless, recent data suggest that ANA could be pathogenic or at least contribute to the perennation of the disease. We review here first the indirect clues of the contribution of ANA to SSc: they are associated to the disease subtypes, they may precede disease onset, their titer correlates with disease activity and severity, there is an association between molecular subsets, and some patients can respond to B-cell targeting therapy. Then, we describe in a second part the mechanisms of ANA production in SSc from individual genetic background to post-transcriptional modifications of neoantigens. Finally, we elaborate on the potential mechanisms of pathogenicity: ANA could be pathogenic through immune-complex-mediated mechanisms; other processes potentially involve molecular mimicry and ANA penetration into the target cell, with a focus on anti-topoisomerase-I antibodies, which are the most probable candidate to play a role in the pathophysiology of SSc. Finally, we outline some technical and conceptual ways to improve our understanding in this field.

## 1 Introduction

The pathophysiology of systemic sclerosis (SSc) is characterized by a triad including autoimmunity, vasculopathy, and excessive fibrosis due to increased extracellular matrix (ECM) synthesis by activated fibroblasts (FB) (1, 2). The humoral immune system plays an important role in SSc (3). Autoantibodies (Aab) are found in almost all patients. Some of these Aab are called functional because of their potential direct role in the pathophysiology of SSc. These Aab may target (a) cell types like anti-endothelial or FB (4, 5); (b) membranous receptors like angiotensin II type 1 receptor, endothelin-1 type A receptor, or platelet-derived growth factor receptor (anti-PDGFR) (6); and (c) ECM components like fibrillin or matrix metalloproteinase-1 (7–9). Yet, these functional Aab are not used in clinical daily practice. On the other hand, anti-nuclear Aab (ANA) are routinely assessed as strong diagnosis and prognosis biomarkers. For example, anti-topoisomerase type I antibodies (ATA) are usually associated with diffuse cutaneous SSc (dcSSc) and the presence, severity, and progression of interstitial lung disease, whereas anticentromere antibodies (ACA) are associated with limited cutaneous SSc (lcSSc) and pulmonary arterial hypertension (1). It is believed that the nuclear nature of their target prevents any accessibility to ANA, precluding any direct pathophysiological role (10). Nevertheless, recent data suggested that ANA could indeed be pathogenic or at least contribute to the perennation of the disease. In this review, we describe the different clues gathered, suggesting the pathogenic role of ANA; then, we try to understand the potential mechanisms of pathogenicity by reviewing the mechanisms of production of Aab and their possible cellular actions.

## 2 Antinuclear Antibodies as Bystanders and Biomarkers in SSc

**Figure 1:**
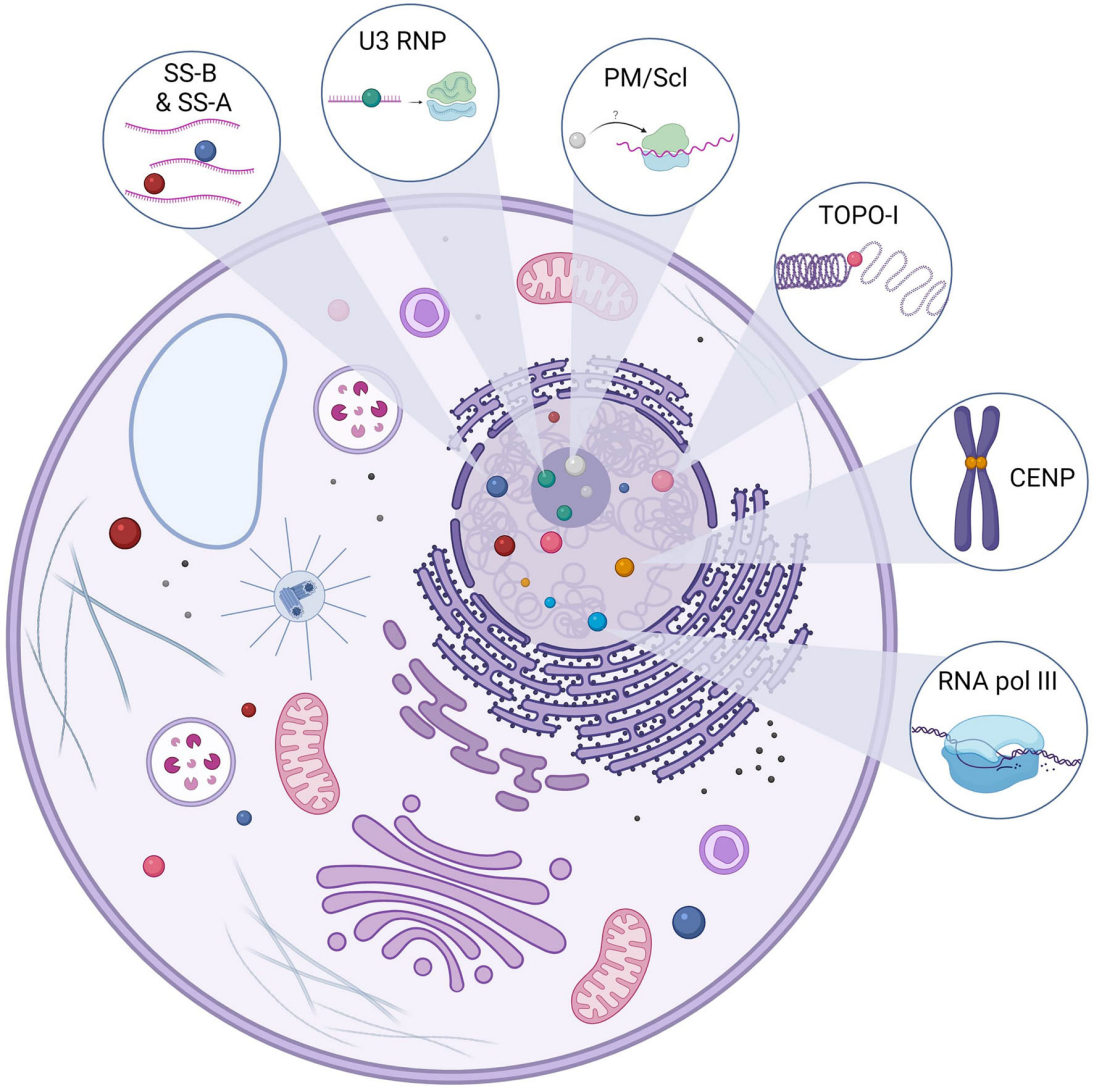
Main antigenic targets of ANA in SSc. Anti-topoisomerase-I autoantibodies (Aab) are specific to systemic sclerosis (SSc). Topoisomerase-I is involved in the DNA relaxation and can be found in the nucleus but also the cytoplasm. There are several centromeric proteins, but three can be targeted by SSc Aab: CENP-A (17 kDa), CENP-B (80 kDa), and CENP-C (140 kDa). CENP-B is the main antigen target, and ACA can recognize other epitopes. Anti-U3 RNP or anti-fibrillarin Aab recognize a 34-kDa protein in the U3-RNP complex in the nucleolus. U3 RNP has a role in the transcription of ribosomal RNA. Pm/Scl is in the nucleolus and is a complex of 11–16 proteins; the 75 and 100 kDa proteins are the main targets of these Aab. Although their precise role remains to be elucidated, they are thought to be involved in the ribosome synthesis. RNA polymerase III is involved in the DNA transcription of RNA and is in the nucleus. Anti-SS-A Aab are directed against two proteins of different molecular masses, namely, 52 and 60 kDa, and those proteins are part of different macromolecular complexes. Ro60 is part of a ribonucleic complex involved in the regulation and transcription of mRNA and Ro52 (also known as TRIM21) and plays a role in the immune response by acting as a cytosolic sensor during viral infection. SS-B or La protein is a protein involved in the maturation of transcripted RNA by RNA polymerase III. The anti-SS-B Aab is always associated with anti-SS-A Aab. Those ANA (SS-A and SS-B) can be found in the nucleus but also in the cytoplasm.

SSc-associated ANA detected in the majority of SSc patients are usually specific [i.e., they are not detected in other systemic autoimmune disease (SAID) or healthy subjects] and mainly mutually exclusive (i.e., only one specificity is found in the same patient during the course of the disease) (11, 12). SSc-associated ANA recognize a wide variety of intracellular targets with variable function and cellular distribution. Intracellular targets of SSc-associated ANA include topoisomerase-I (TOPO-I), centromeric proteins (CENP), polymerase enzymes such as RNA polymerase III (RNAP), ribonuclear proteins (U3 RNP/anti-fibrillarin, U1 RNP, U11/U12 RNP), or less frequently nucleolar antigens (Th/To, SS-A, and SS-B, NOR 90, Ku, RuvBL1/2, and PM/Scl) (11). Putative antigenic targets in SSc are described in **Figure 1**. TOPO-I is a 100-kDa nuclear enzyme responsible for DNA relaxation during transcription (13). ATA frequency in SSc varies from 8% to 42% (11, 14). ATA are highly specific of SSc and are associated with a more severe SSc with an extensive and worsening skin and lung fibrosis (15). ATA are independent worse prognosis biomarkers for survival (16–18).

Centromeres are the site of chromosome that assemble the kinetochore, which permit safe cellular division (19). In SSc, ACA target either centromeric chromatin (CENP-A and CENP-B) or kinetochore (CENP-C) (20). ACA frequency in SSc varies from 20% to 40%, and they have been detected in other SAID (primary Sjogren’s syndrome, systemic lupus erythematosus, rheumatoid arthritis, and primary biliary cirrhosis) (21–23). ACA are associated with lcSSc, pulmonary arterial hypertension, and a vascular phenotype (24).

Anti-RNA polymerase III antibodies (anti-RNAP) are detected in 12% of SSc patients and are associated with dcSSc, rapidly progressive skin extension, renal crisis and co-occurrence of cancer (11, 25–27). These three ANA subtypes (ATA, ACA, and anti-RNAP) permit to classify the majority of SSc patients and have been included in the last 2013 American College of Rheumatology/European League Against Rheumatism classification (28). Patterson et al. analyzed ANA (using commercial immunoassay containing 10 ANA specificities) of 505 Australian SSc and performed hierarchical clustering of the first 2 dimensions of a principal components analysis of quantitative Aab scores. Principal component analysis clustering demonstrated mutually and exclusive relationships between ATA, ACA, and anti-RNAP. Five clusters of patients were found: ACA, ATA, anti-RNAP strong positive response, anti-RNAP weak positive response, and others. These data reinforced the clinical associations between ATA, ACA, and the phenotypes previously described. Moreover, they highlighted two clusters of anti-RNAP with distinct clinical forms: patients in the anti-RNAP strong cluster had an increased risk of gastric antral vascular ectasia, but a lower risk of esophageal dysmotility, whereas patients in the cluster anti-RNAP weak were more likely to be male and to have a history of malignancy (29). In addition, cluster analysis based on 24 clinical and serologic variables performed in 6,927 SSc patients revealed six homogeneous groups, which differed with regard to their clinical features, ANA profile, and mortality, with distinct prognosis. This clustering revealed that classification based on skin involvement is not sufficient to explain SSc heterogeneity and that heterogeneity is partially explained by ANA subsets (16). By combining Aab specificity and extent of skin involvement in the Royal Free cohort, Nihtyanova et al. proposed in a recent study a novel SSc classification scheme including seven groups (ACA lcSSc, ATA lcSSc, ATA dcSSc, anti-RNAP, anti-U3RNP, other antibodies lcSSc, and other antibodies dcSSc), which enabled a more precise risk stratification of patients than the classical cutaneous subsets (30). Hence, SSc-associated ANA are mutually exclusive, stable over time, and strongly linked to the phenotype of patients, which is troubling and suggests a role in the mechanisms of the disease.

## 3 Indirect Proofs of Pathogenicity

In addition to being relevant biomarkers, several findings seem to point to a pathogenic role of ANA. First, the presence of these markers may precede the disease by several years despite any clinical manifestation. A case–control study of 46 SSc patients (16 with renal involvement and 30 without) revealed that 75% of patients with renal crisis and 40% without renal crisis were seropositive for at least one Aab prior to clinical diagnosis (up to 27.1 years earlier, mean = −7.4 years). The positivity for ANA before disease onset was found for anti-RNAP, ATA, and anti-SS-A (52 and 60 kDa). Interestingly, the authors found that the total response against the autoantigen panel harbored higher Aab levels in the cohort of patients with renal crisis (31). Koenig et al. performed a prospective study on 585 individuals with Raynaud’s phenomenon. They found that patients with positive ANA and nailfold capillary microscopy anomalies at baseline develop definite SSc in 80% of the cases. ACA and anti-Th/To predicted enlarged capillaries and anti-RNAP predicted capillaries loss, which linked these ANA to the occurrence of a vascular phenotype. This study highlighted that ANA could be present years before the onset of SSc manifestations besides the Raynaud’s phenomenon (32). These data are consistent with the analysis of 130 US veterans’ ANA sera collected prospectively before they received a diagnosis of another SAID, i.e., systemic lupus erythematosus (SLE). They found that ANA, anti-Sm, and anti-DNA Aab were present years before disease onset, which indicate that ANA accumulate when patients are asymptomatic and predicted SLE onset (33). Furthermore, in patients with undifferentiated connective tissue disease, the presence of ATA or ACA is independently predictive of SSc development (34, 35). In SSc, the concept of very early diagnosis of SSc (VEDOSS) emerged. VEDOSS criteria were defined by the presence of Raynaud’s phenomenon, puffy swollen digits turning into sclerodactyly, abnormal capillaroscopy with scleroderma pattern, and SSc-specific ANA positivity (ATA or ACA) (36). Finally, Aab against various peptides of TOPO-I persist after autologous stem-cell transplantation, and their reactivity were higher in patients who relapse after autologous stem-cells transplantation, which point a potential involvement of ATA in disease activity (37).

Second, it has been shown that ANA titers vary among time and patients, which may explain phenotype and severity differences between SSc with the same ANA subtype. For example, higher levels of ATA are correlated with metacarpophalangeal and proximal interphalangeal involvement (38). Based on IgG ATA levels measured by enzyme-linked immunosorbent assay (ELISA) on 182 SSc patients at first visit, Perera et al. showed that IgG ATA levels correlated with skin thickness progression (39). Furthermore, higher levels of IgG or IgA ATA measured by ELISA were positively correlated with total skin score and disease activity score (40). A few data are available on IgM Aab titer and disease activity. IgM ATA could be found in about 20% of SSc patients (41). In ATA-positive patients, the presence of IgM ATA and a higher titer of IgM ATA at baseline is associated with disease progression (42). Moreover, disappearance of ATA during the disease has been associated with a favorable outcome (43) and re-emergence of ATA associated with skin exacerbation (44). Finally, frequencies of TOPO-I-reactive CD27+ B cells are increased in ATA-positive patients compare to ACA-positive patients and healthy subjects. Yet, to our knowledge, there are no data in the literature concerning the follow-up of TOPO-I-specific B cells in ATA-positive patients (45). Same results have been found about ACA-positive patients. Recently, in a large cohort of VEDOSS and definite SSc, titers of IgG and IgM ACA were higher in the group with definite SSc. Follow-up of very early SSc revealed that progression to definite SSc was associated with higher IgG ACA levels at baseline (46).

Third, SSc patients may improve after immunomodulatory therapy. Intravenous immunoglobulins (IVIg) are known to modulate Aab action in various autoimmune diseases (47, 48). In SSc, IVIg could be effective by reducing skin involvement, mostly in ATA-positive patients, and may be an option for associated myositis (49–52). B-cell depletion have been evaluated with anti-CD20 therapeutic such as rituximab (RTX) (53). A pilot study of RTX on 15 dcSSc patients showed no statistical improvement of skin fibrosis or lung involvement but revealed a reduced sera and skin B-cell number and reduced skin myofibroblast number, while having little effect on Aab levels (54). RTX seemed to have beneficial effects on skin involvement and disease activity score in early dcSSc (55). A controlled double-blind trial vs. placebo confirmed the improvement of SSc skin in the RTX group (56). Finally, a retrospective study comparing cyclophosphamide treatment vs. RTX in ATA-positive dcSSc patients with interstitial lung disease showed a better improvement in forced vital capacity, diffusing capacity of the lung carbon monoxide, and mean Rodnan skin score in the RTX group (57).

Finally, a link between molecular transcriptomic patterns and ANA subtypes is emerging. Inamo et al. explored skin gene expression profiles by microarrays in different ANA subtypes. This study showed that some genes expression, such as the *integrin subunit beta 5* (ITGB5) gene or the *actin alpha 2* (ACTA2) gene, were commonly overexpressed in patient seropositive either for ACA, ATA, or anti-RNAP. On the other hand, conducting a functional enrichment analysis on skin genes expression profiles, they found a strong correlation between functional enrichment profiles and ANA serotypes (58). Interestingly, validated serum biomarkers of skin fibrosis (such as type III procollagen peptide, hyaluronic acid, and metalloproteinases 1) differed according to the ANA subtype in early SSc, especially between ATA and anti-RNAP. In this study, RNA sequencing of whole skin biopsy from dcSSc patients discriminated between ATA- and anti-RNAP-positive patients (59).

In summary, ANA can precede the disease, may correlate with disease activity, and are associated with skin transcriptome patterns. The efficacy of IVIg or B-cell targeting therapy is another argument favoring the potential pathogenic role of ANA. Clues of pathogenicity of some ANA are represented in **Table 1**.

**Table 1:** Clues of ANA pathogenicity in SSc.

ANA subtype	Specificity to a distinct clinical subset	Present before disease onset	Titers correlate with phenotype and severity	Association with molecular subset	Response to immunomodulatory drugs
ATA	+++	++	+++	+++	++
ACA	+++	++	++	++	+
Anti-RNAP3	+++	?	?	+++	?

ATA, anti-topoisomerase-I antibodies; ACA, anticentromere antibodies; anti-RNAP, anti-RNA polymerase III antibodies. +++: high level of evidence; ++: medium level of evidence; +: low level of evidence.

## 4 Challenges to the Comprehension of the Pathogenic Role of ANA in SSc

Pathogenic Aab are usually defined as immunoglobulins contributing to the development of an autoimmune disease and its organ manifestations. This definition was specified by Naparstek and Plotz, who stated that any pathogenic Aab should be found in combination with a plausible antigen at the site of tissue damage and should cause the lesions attributed to it in an experimental setting (60). Proofs of pathogenic Aab are usually established when the transfer of these Aab induces the disease (human to human transfer or human to animal transfer) or when, *in vitro*, these Aab induce apoptosis or modify the target cells (61). Some of these pathogenic Aab can be further specified as functional if they directly activate or inhibit a molecular pathway (25). These definitions are particularly true in specific organ auto-immune diseases mediated by Aab like pemphigus, thyroiditis, or autoimmune encephalitis in which the autoantigen is membranous and thus accessible to Aab. In SSc, the nuclear nature of the ANA targets theoretically prevents any accessibility, making the proof of their potential pathogenicity difficult to establish. There are some conceptual and technical issues to overcome in the path of understanding the possible pathogenic role of Aab in SSc:

- ANA production: which mechanisms underpin ANA generation from genetic background to post-transcriptional modifications of neoantigens? ANA production implies a source of self-antigen or neoantigen and a breach in tolerance (62, 63).
- Are SSc ANA able to cause disease after passive transfer? Disease induction after Aab passive transfer in animal models is usually admitted as a proof of pathogenicity, which is not yet proven for ANA in SSc (61, 64).
- Aab used for experiments: the first step is to decide to use whether total IgG from patients (containing ANA, maybe functional Aab and others IgG) or specific purified IgG (for example, ATA, ACA, or anti-RNAP). It is also crucial to determine which isotype of Aab to study (IgG, IgM, or IgA) (42).
- Which is the precise antigenic target? Since ANA target nuclear antigen, it is essential to answer the question of the precise antigen recognized. (i) Are they able to recognize another antigenic target like membranous antigen (65)? (ii) Are they pathogenic through immune complexes (ICs) (66)? (iii) Are they effective through their common region (*via* Fc gamma receptor) or *via* Fab’ fragment (67)? In the latter case, do they recognize membranous antigen or are they able to reach the nucleus to interact with their nuclear target?
- Which cell type are targeted by ANA? ANA could be pathogenic for example by directly activating the FB and thus inducing a fibrotic process or by the effect on the endothelial cell. Moreover, the choice of the target cell exists within the same cell type. Concerning the dermal FB, they may come from healthy subjects’ skin, unaffected SSc skin, or affected SSc skin. The last two raise the problem of the heterogeneity of FB in SSc, which may complicate the experiment (68).
- Which is the fraction of pathogenic Aab among a particular subtype. For the same group of ANA, the reactivity towards the epitopes is heterogeneous (69). Thus, the Aab response to an antigen is probably polyclonal and only a fraction of these Aab may be pathogenic.

To answer these questions, we develop here these different points according to the literature existing in SSc.

## 5 ANA Generation

Production of pathogenic Aab implies a substantial breach in tolerance to self-antigens and a source of self-antigens or neoantigens (70). Thus, most of neoantigens are localized in the nucleus in SSc, and it seems relevant to explore the mechanisms behind the self-tolerance breach and ANA generation to explore their pathogenic implication. ANA generation in SAID should imply release of neoantigens by apoptotic blebs, epigenetic and post-translational modifications (PTM), molecular mimicry, and dysregulation of immune response (71). This could result in a polyclonal response toward neoantigens. For example, an autoantigen study of 40 kinetochore macro-complex antigens in ACA-positive SSc patients revealed that sera recognized several antigens of the centromere complex (20).

### 5.1 Apoptotic Blebs

Apoptotic blebs are known to be a source of neoantigens in SAID as described in SLE in which accumulation of modified self-antigens during apoptosis leads to self-immunization (72–74). In SSc, apoptotic blebs of endothelial cells are described on skin biopsies from patients at early stage of the disease and mononuclear apoptosis cells in the skin of bleomycin-induced SSc mice model (75, 76). Furthermore, TOPO-I released by apoptotic blebs induced *in vitro* pro-inflammatory and pro-fibrotic effect on FB (77).

### 5.2 Post-Translational Modifications of Neoantigens

PTM can generate neoantigens and lead to autoimmunity (62). The oxidation of TOPO-I antigen is sufficient to break the tolerance in mice, induce the production of ATA, increase FB proliferation, and increase type I collagen mRNA expression. In this model, oxidation of TOPO-I by H_2_O_2_ creates modified neoepitopes, which can promote ANA production (78). Moreover, oxidation of TOPO-I by “Fenton's reaction” alters the antigen and increases the reactivity towards the ATA (79). Other PTM modifications are described. Sera from SSc patients contain IgG antibodies targeting distinct sulfated carbohydrates, suggesting that specific PTM carbohydrate modifications may act as an important immunogens source in SSc (80). Carbamylation is a PTM modification that affected proteins with low turnover like dermal proteins. IgG against carbamylated proteins were detected in SSc and correlated with skin fibrosis (81). However, there is no correlation with carbamylation and specific SSc ANA subtype. Thus, PTM of the antigen may increase the immunological response against it and generate Aab. Then, by an epitope spreading mechanism, Aab could target both the modified antigen (the oxidated TOPO-I) and the normal antigen (non-oxidated TOPO-I).

### 5.3 Environmental Factors

Several environmental factors like silica and solvents are described as etiological agents of SSc (82). Crystalline silica impaired human-monocyte-derived macrophages and mouse pulmonary macrophage apoptotic cells clearance (83). Genetic factors are described in silica-associated SSc, and ATA is the predominant associated Aab (84, 85). These data indicate that in genetically predisposed individuals, environmental factors promote impairment of apoptotic blebs clearance and therefore the release of neoantigens leading to ANA production.

### 5.4 Molecular Mimicry and Epitope Spreading

A commonly accepted hypothesis is that SAIDs originate from a molecular mimicry mechanism leading to immunization by epitope spreading. The hypothesis of molecular mimicry makes the link between infectious disease or cancer and auto-immune diseases. The relationship between viral infection and SSc ANA has been discussed for years. For example, some IgG Aab that react with SSc antigens can bind human cytomegalovirus late protein UL94. These Aab that reacted with both SSc antigen and viral peptide are able to bind to endothelial cells and induce apoptosis (86). We develop here some evidence of molecular mimicry, which could be a source of ANA in SSc.

#### 5.4.1 ACA

After identifying the autoantigenic motif of CENP-A responsible of the immune response, Mahler et al. (using immobilized nucleotide arrays) found that the epitope of this motif is present in a vast number of autoantigens and in the Epstein–Barr virus nuclear antigen 1. They then performed affinity purification of Aab against the identified peptide. These Aab were polyclonal and cross-reacted with others autoantigen (most often with cryptic epitopes) like CENP-B or KU-80. In this study, all sera from ACA-positive patients were positive for anti-EBV antibodies (87). The possibility of molecular mimicry in the generation of ACA seems interesting and deserves further studies.

#### 5.4.2 ATA

Bioinformatic analysis revealed that many pathogenic agents share amino acid homologies with autoepitope from TOPO-I and CENP-A. Nevertheless, it remains unclear how peptides are targeted with cross-reactive ANA present in patients and thus the role of pathogens in ANA generation (88). Gourgh et al. predicted immunodominant peptide of self-antigen according to ANA subtype by studying HLA gene. Then, they were able to explore homology sequences between this predicted immunodominant peptide and viral protein sequences from Mimiviridae and Phycodnaviridae families. For example, one sequence of TOPO-I highly matches with a peptide from Mimiviridae family (89). Altogether, these data support the hypothesis that SSc-associated ANA may arise through molecular mimicry.

#### 5.4.3 Anti-RNAP3

As discussed above, anti-RNAP3 are strongly associated with the presence of cancer in SSc. Immunochemistry analysis of tissue cancer from anti-RNAP-positive patients revealed a strong RNAP3 staining in the tissue. These data support the idea that cancer initiates specific immune response and drives to a clinical phenotype of SSc (90). High-throughput epitope identification method did not detect difference between anti-RNAP3 patients with or without cancer. The same study demonstrated the extent of intramolecular spreading among RNAP complex and no correlation with the presence of cancer. Thus, immune response could be initiated by one component of the macro complex and spread to its other epitopes (91). Taken together and since anti-RNAP3 are also present without neoplasia, we can hypothesize that tumoral cells initiate immune response by exhibiting mutated RNAP3. This response spreads to other components of RNAP3 without mutation and leads to the SSc phenotype. Then, cancer persists or regresses, thanks to the immune response (92).

### 5.5 Dysregulation of Immune Response

B-cell (3, 93, 94) and T-cell (95, 96) homeostasis is impaired in SSc. CD11a regulates adhesive and co-stimulatory interactions between CD4+ T cells and other cells. Wang et al. showed that CD11a was overexpressed and hypomethylated on CD4+ T cells from SSc patients compared to healthy subjects. In addition, there was a higher production of total IgG in B cells co-cultured with autologous CD4+ T cells from SSc patients than with CD4+ T cells from healthy subjects (97). These data indicate that immune dysregulation could contribute to ANA generation in SSc.

**Figure 2:**
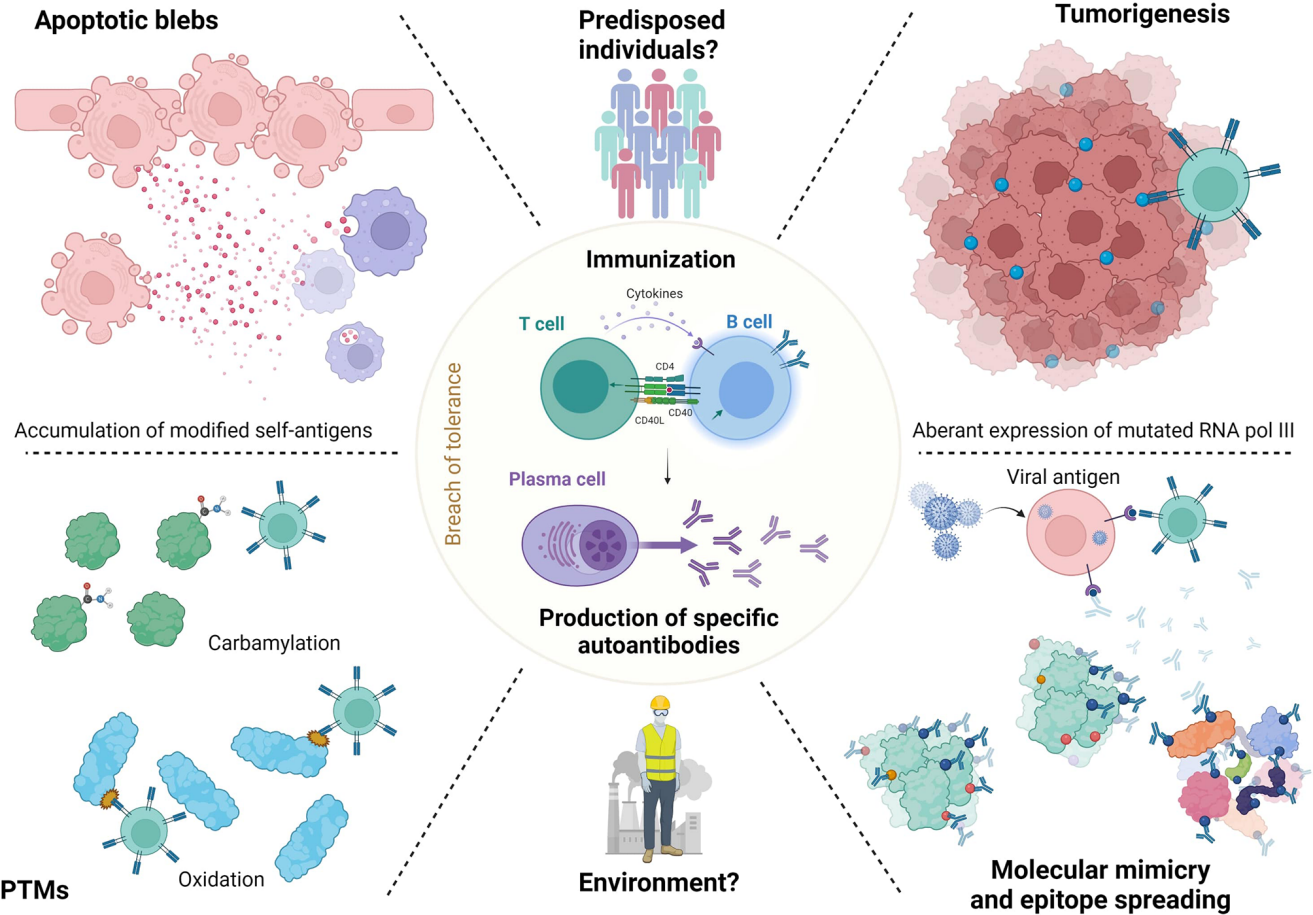
ANA generation in SSc. Release of neoantigens could lead in predisposed individuals to immunization and antibodies generation. These neoantigens may raise from apoptotic blebs. Their release could be favorized by environmental factors like silica or solvents. Neoantigens can undergo post-translational modification (PTM) or modifications by tumorigenesis (RNA polymerase 3). Then, epitope spreading mechanisms lead to immunization to both modified or normal antigens, which generate antibodies. Finally, molecular mimicry mechanisms with viral agents like Epstein Barr virus are also described.

To conclude this part, we can hypothesize that altered neoantigens are released by apoptotic cells or overexposed by neoplastic cells or by virus, become dominant, and trigger specific immune responses through predisposed T and B cells. The mechanisms of ANA generation in SSc are summarized in **Figure 2**.

## 6 Basic Proof of ANA Pathogenicity in SSc and Possible Mechanisms Involved

### 6.1 Induction of Disease by Antibodies

The formal evidence of pathogenic Aab is established when the passive transfer of Aab induces the disease. Several models are used to prove induction of disease by Aab: transplacental, human to human, human to animal, and animal to animal transfer. The passive transfer of IgG from SSc patients to animal has been studied for functional Aab. Anti-myenteric (98), angiotensin II type 1 receptor, anti-endothelin-1 type A receptor (99), and anti-PDGFR (100) induced pathogenic features in mice models after transfer. Concerning ANA, Mehta et al. immunized bleomycin mice with TOPO-I loaded in dendritic cells. This immunization induced specific SSc Aab and skin and lung disease (101). Moreover, immunization of mice with TOPO-I and Freud adjuvant induced ATA antibody generation, IL-6 production, and skin and lung fibrosis. IL-6 deficiency ameliorated skin and lung fibrosis induced by TOPO-I, suggesting that fibrosis in this immunization model is IL-6 dependent. These data are consistent with the role of IL-6 in SSc (102, 103). Nevertheless, to date, there are no models of ANA passive transfer in SSc.

### 6.2 Immune Complexes and ANA/Membranous Antigen Interaction

ANA may be pathogenic through IC containing ANA and nuclear autoantigen. Besides, it is admitted that the pathogenic role of Aab is amplified when autoantigens are accessible to IC formation (104). Some studies have found the presence of IC in the serum of about 40% of SSc patients (105–108), and IC are also found in the bronchoalveolar lavage fluid of SSc patients with interstitial lung disease (109). To our knowledge, the deposition of IC in the tissue (which is the main mechanism of pathogenic role of IC in other SAID like SLE) has not been established in SSc (110). However, heavy and light chains of immunoglobulin mRNA are found in whole skin transcriptomic of SSc patients (111). The hypothesis of pathogenic IC seemed interesting, since some of the purified ATAs are able to interact with compound at FB membrane (112). Hénault et al. explored the membrane antigenic target of ATA on FB and human umbilical vein endothelial cells. They showed that TOPO-I from apoptotic cells bound to the FB membrane and are recognized by ATA. TOPO-I did not bind to human umbilical vein endothelial cells (77). Nevertheless, the consequences of the binding of ATA were not explored.

### 6.3 IC and FB/Fibrosis

**Figure 3:**
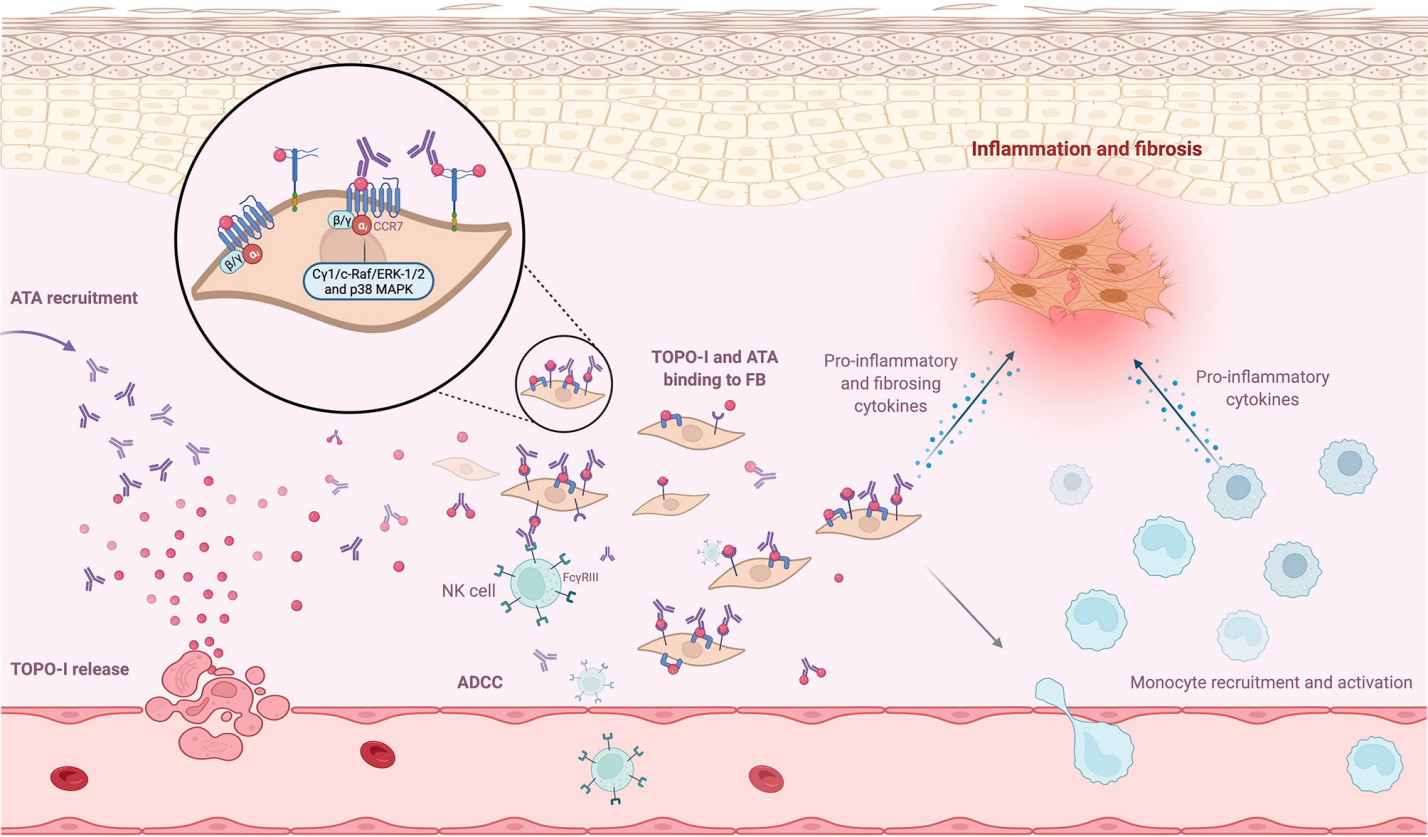
ANA and antigen interaction at cell surface. Neoantigens, such as topoisomerase-I (TOPO-I), are released by apoptotic blebs. TOPO-I bind to CCR7 or proteoglycans at the membrane and are recognized by anti-topoisomerase-I (ATA). This binding recruits ATA at the FB membrane and promotes inflammatory and profibrotic pathways. The recruitment of more ATA activates monocytes and could promote autoantibody-dependent cell-mediated cytotoxicity mechanisms.

Arcand et al. explored TOPO-I-mediated cellular effects and characterized the specific target of TOPO-I on FB. They found that (i) IgG from ATA-positive patients amplified the binding of TOPO-I to normal dermal FB membrane; (ii) TOPO-I antigen binds to the FB membrane by interacting with heparan sulfate proteoglycan; and (iii) heparin inhibits the binding of TOPO-I and IgG ATA+/TOPO-I IC to the heparan sulfate proteoglycans at the membrane of FB. This binding correlated with the titer of anti-TOPO-I (113). This result suggested that the topo-I antigen gene could be overexpressed in SSc FB from affected and unaffected skin (114). The same team also demonstrated that TOPO-I binds to FB surface *via* CCR7 and activated intracellular signaling pathways (Cγ1, c-Raf, ERK-1/2, and p38 MAPK) that stimulated FB migration *via* a G(αi) protein-coupled receptor (115). Moreover, Hénault et al. demonstrated that TOPO-I binding to the FB surface recruits ATA and induces adhesion and activation of monocytes (77). Interestingly, a genotype study showed a functional FCGR3A-V158F polymorphism in ATA-positive patients, suggesting autoantibody-dependent cell-mediated cytotoxicity (ADCC) (116). This high-affinity FCGR3 polymorphism on ATA-positive patients cells (such as natural killer cells or monocytes) could enhance autoantibody-dependent cell-mediated cytotoxicity (ADCC) or proinflammatory cytokines production in the presence of a low amount of deleterious ATA (117, 118). To summarize, TOPO-I is released by endothelial apoptotic cells in the tissue and binds to FB membrane, since TOPO-I has a high affinity for FB membrane. This binding might have two implications: inducing proinflammatory and profibrotic properties of FB and recruiting more ATA, which then activate monocytes and could promote ADCC mechanisms. **Figure 3** represents the mechanisms promoting FB activation after binding of TOPO-I to its membrane.

Raschi et al. purified IC containing SSc-associated ANA. FB from SSc skin were incubated with these specific IC. Interestingly, FB expressed different fibrosis, inflammatory profile, and pathways according to ANA subtype containing in IC. For example, Th/To-IC response was preferentially mediated by p38MAPK, whereas ATA-IC, ACA-IC, and ARA-IC engaged nuclear factor kappa B (NF-κB)/p38MAPK. Moreover, the authors suggested that these findings may be mediated by the interaction with the different Toll-like receptors (TLRs) at the FB membrane and part of the autoantigen composed of nucleic-acid embedded in IC. The different ANA/neoantigens complexes engaged different TLR responses. In this study, TLR2, TLR3, and TLR4 were engaged on FB by ATA-IC and ACA-IC, whereas TLR9 were engaged by Th/To-IC (119). Membranous TLR2 and TLR4 and endosomal TLR3 and TLR9 are expressed on FB from SSc patients (120). Moreover, TLR ligands (such as serum amyloid A for TLR2 and tenascin C for TLR4) are increased in SSc sera and can activate FB (120–122).

### 6.4 Endothelial Cells and IC

**Figure 4:**
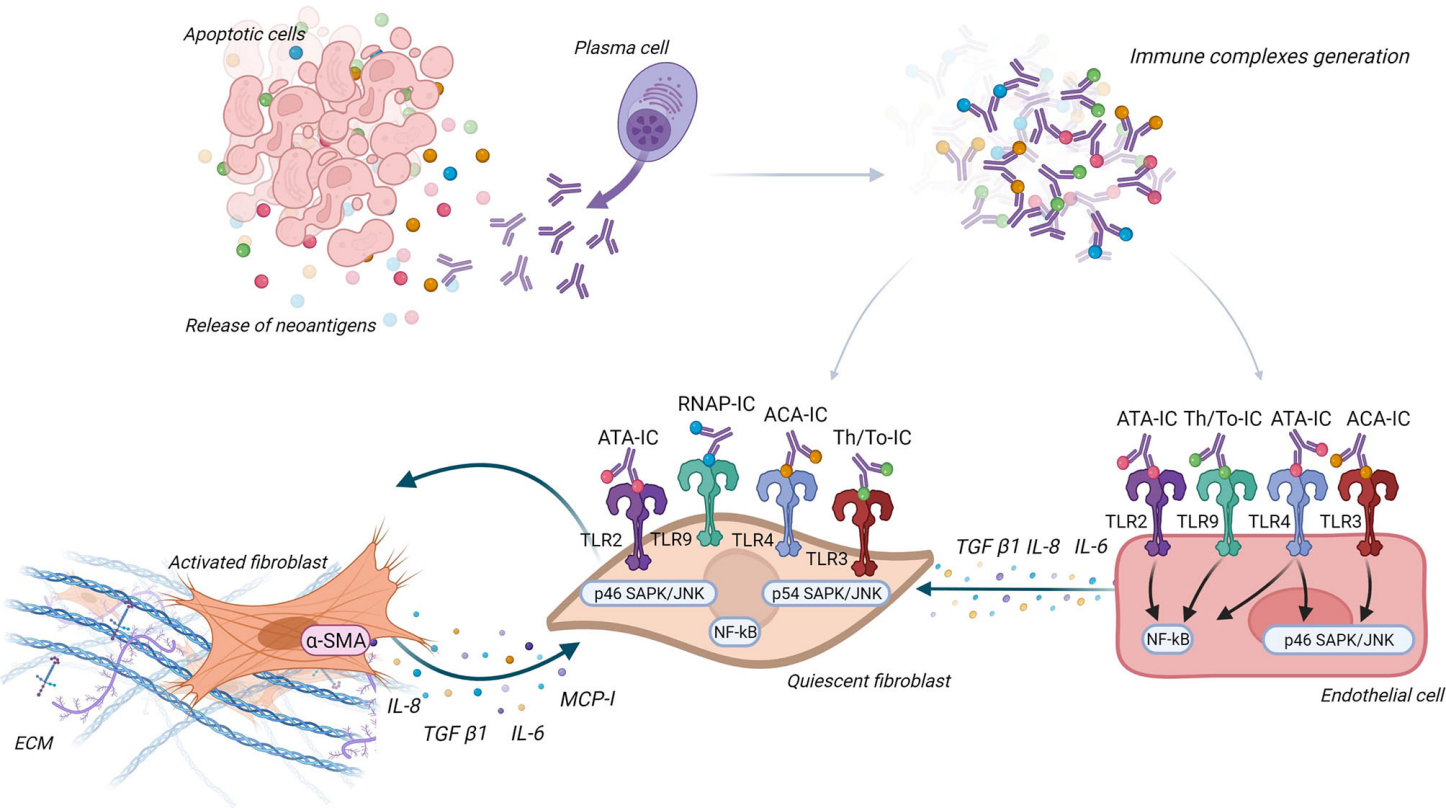
Immune complexes theory. Antinuclear antibodies (ANA) interact with their neoantigens released by apoptotic blebs and form immune complexes (IC). These IC interact with different Toll-like receptors (TLR) according to ANA subtypes, both on fibroblast (FB) and endothelial cells. FB are then activated into myofibroblasts and secrete extracellular matrix and proinflammatory and profibrotic cytokines. Endothelial cells engage pathways according to ANA subtype (like NF-κB and SAPK/JNK) and secrete cytokines that activate FB.

IgG being in plasma, it seems important to study the interaction between IgG, endothelial cells, and FB to better understand the interactions between Aab, vasculopathy, and tissue fibrosis. An *in vitro* study suggested that IC may induce antibody-dependent cellular cytotoxicity on endothelial cells through mononuclear cells and FcγR (123). Raschi et al. also demonstrated that IC containing specific SSc ANA induce vascular damage through the same TLR mechanism as described above. Moreover, FB and endothelial cells co-culture revealed that endothelial cells are able to secrete cytokines like IL-6 or transforming growth factor-β (TGF-β) after the interaction between nucleic acid contained in IC and TLR, this leading to FB activation (124). **Figure 4** represents the different ways IC could contribute to pathogenesis *via* activation of FB and endothelial cells.

### 6.5 IgG Direct Role on Target Cells

Corallo et al. assessed the direct pathogenic role of ATA and ACA on FB from healthy subjects and affected and unaffected dermal FB from lcSSc and dcSSc. Polyclonal ATA and ACA increased fibrotic markers (mRNA: *ACTA2*, *COL1A1*, and *TAGLN*; and proteins: α-SMA, COL1A1, and TGLN) especially when incubated on unaffected lcSSc and healthy controls FB. However, these profibrotic markers levels were higher in affected lcSSc and dcSSc FB at basal conditions, i.e., without IgG incubation (125). Similarly, Shen et al. showed that sera containing ACA and ATA from SSc are able to induce *in vitro* endothelial cells senescence (126). These data suggested an interaction with target cell membrane through epitope/paratope interaction. In the case of epitope/paratope interactions, ANA could recognize their antigenic epitope expressed at the membrane (as discussed above: interaction with ATA and TOPO-I at the membrane of FB) or recognize other antigenic targets at the surface of the target cells, with functional properties. For example, in SLE, native anti-DNA Aab have been described as able to recognize other targets (actinin or annexin-2) in mesangial cells by cross-reactivity and induce kidney damage (65, 127, 128).

### 6.6 Penetration Into the Cells

It is commonly accepted that ANA do not penetrate living cells. Nevertheless, there is little evidence that ANA can indeed penetrate the cells, reach their antigenic targets, and induce cellular processes that result in pathogenic features. Mechanisms involved in intracellular penetration of ANA are dependent or independent of FcγR.

For example, the first evidence of cell entrance of Aab targeting intracellular target was made for Aab targeting nuclear ribonucleoprotein, which may be able to penetrate viable human mononuclear cells, by their surface FcγR, and react with nuclear ribonucleoprotein (129).

The mechanisms independent of FcγR for Aab endocytosis are described, especially in SLE. In SLE, the administration to normal mice of a subset of monoclonal anti-DNA induced nuclear deposition on multiple cell types of several organs. In kidney, anti-DNA internalization was associated with glomerular hypercellularity and proteinuria. Nuclear localization was obtained after injection of F(ab)′2 fragments of these anti-DNA antibodies, suggesting an FcγR-independent mechanism. The hypercellularity and proteinuria were not associated with complement deposition and cellular or platelet infiltration, suggesting that the morphological and functional abnormalities were mediated by a direct effect of intranuclear antibodies within glomerular cells (130). An *in vitro* study using confocal microscopy demonstrated that anti-DNA Aab were initially observed at the cell surface, then within the cytoplasm, clustered at the nuclear pore and finally within the nucleus. Furthermore, some of these Aab were recycled back to the cell surface and could serve as a neoantigen for immune deposit formation (131). After binding to calreticulin at the membrane, these anti-DNA interact with DNAse 1 into the cytoplasm (132, 133). These subpopulations of anti-DNA Aab co-localized with myosin 1, shortly after internalization, within caveolae, near the cell membrane (132, 134). This Aab internalization induce an inhibition of P53, thus an inhibition of apoptosis and may contribute to proteinuria (135). The endocytosis of Aab also may occur by caveolae/lipid raft endocytosis (136), electrostatic interactions of arginine residues in the complementarity-determining regions with the negatively charged sulfated polysaccharides on the cell surface (137), or by interaction with glycosaminoglycans (138).

**Figure 5:**
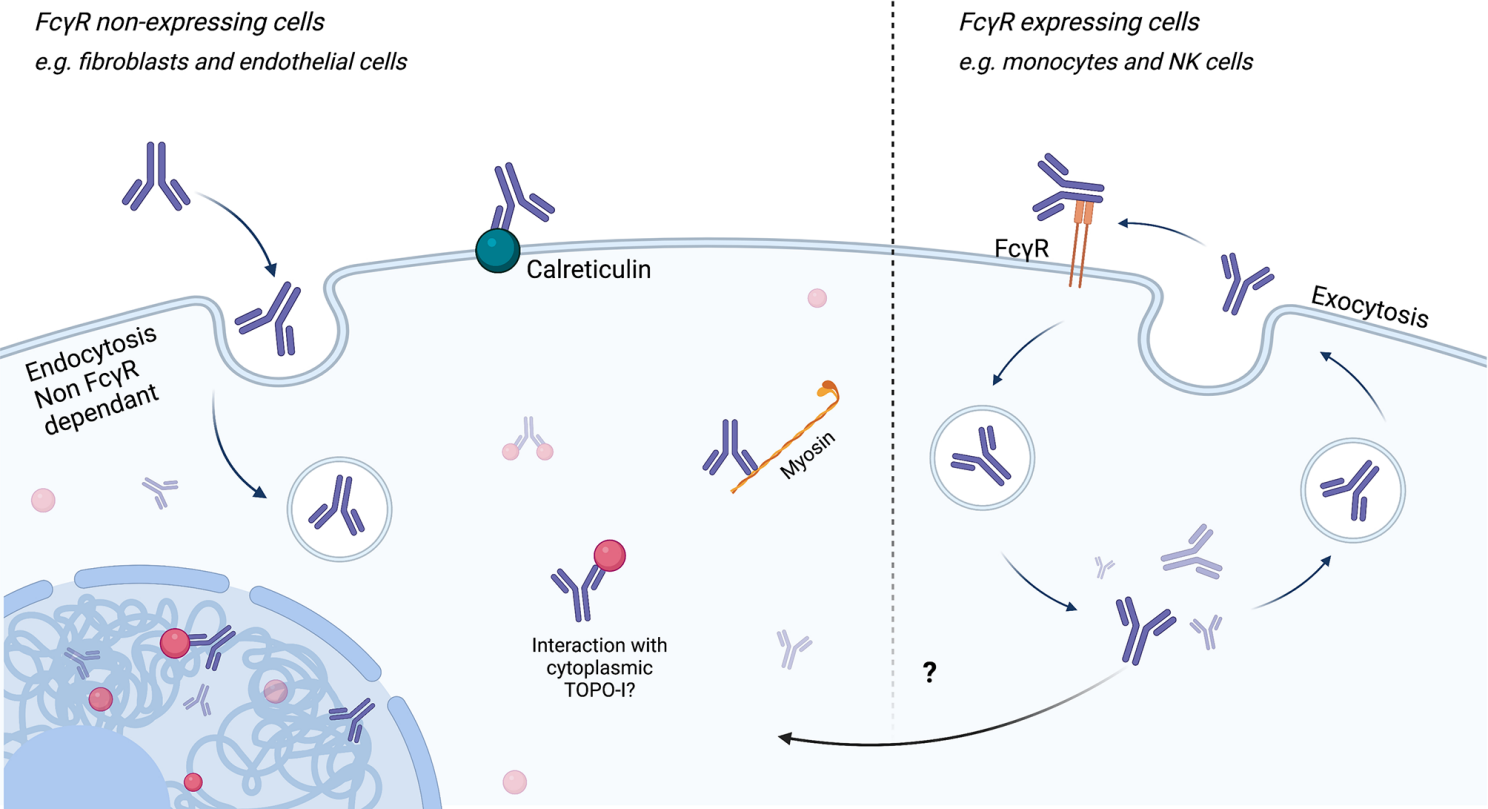
Endocytosis/cell penetration of ANA. Endocytosis of antinuclear antibodies (ANA) can be achieved *via* FcγR-dependent or FcγR-independent (for example, caveolin pathways) mechanisms. Endocytosis independent of FcγR could be carried out through binding to calreticulin at the membrane and then myosin in the cytoplasm. After reaching the cytoplasm or the nucleus, ANA may interact with its antigen and induce cell modifications.

Using confocal microscopy, Ronda et al. showed that purified IgG anti-fibroblasts Aab (AFA) from SSc patients, negative and natural IgG, are able to bind to the FB membrane. Only AFA IgG were internalized into the normal and pathological FB. This penetration was not affected during co-incubation by IgG from healthy subjects, suggesting a mechanism specific to these Aab. Intracellular AFA-positive IgG colocalized with caveolin and the internalization were completely inhibited in the presence of filipin, which disassembled the FB caveolae. Since FB seem to not exhibit FcγR, authors concluded that AFA in SSc patient sera interact with specifically expressed membrane molecules on FB, *via* an FcγR-independent mechanism (139). About half of AFA-positive patients are also positive to ATA. TOPO-I antigen is overexpressed in interstitial lung disease and overexpressed in FB from patients with SSc and is upregulated by TGF-β and tumor necrosis factor alpha (TNF-α) (140, 141). Interestingly, FB from SSc patients cultured with non-cytotoxic dose of camptothecin (a TOPO-I inhibitor) showed a decreased mRNA expression of collagen and deposition of collagen compared to SSc without camptothecin treatment and heathy FB with camptothecin treatment (142). Thus, we can then imagine that the ATA directly modulate the activity of TOPO-I by interacting directly with it in the cells. More recently, ACA obtained after mice immunization with CENP-A and CENP-B were able to penetrate into living early-stage mice embryo (143). Nevertheless, the hypothesis of intracellular internalization of Aab in SSc is only touched upon at present. **Figure 5** represents the different ways of ANA penetration into cells.

## 7 Current Research Gaps in the Field

We have shown so far in this review indirect (strong association between Aab and phenotype and clinical prognosis) and basic evidence of the pathogenicity of ANA in SSc. The latter gives some interesting clues, but these studies had limitations to fully answer the question.

### 7.1 Antibodies

Most of the studies explored the pathogenic effect of total purified IgG or sera containing ANA. Nevertheless, we cannot exclude the presence of functional Aab (such as anti-PDGFR or anti-endothelial cells Aab) in sera or total IgG, which have effects on target cells. It would therefore be useful to study the role of specifically purified SSc ANA. Moreover, SSc ANA are probably polyclonal, and it is possible that only certain clones have pathogenicity. Finally, some PTM of IgG like glycosylation of Fc fragment are described to promote pathogenicity in other systemic autoimmune disease, which is not fully explored in SSc in our knowledge.

### 7.2 Technical Issues

The techniques used so far may not be sufficient to establish the pathogenicity of ANA on target cells (such as FB or endothelial cells). For example, most of the studies focused on specific pre-chosen markers (like collagen mRNA for fibrosis) inducing a bias in the interpretation of the results. More sensitive and non-targeted approaches such as multiomics studies are needed to fully understand this research field (144).

### 7.3 Mechanism and Specific Pathways Involved

The precise interaction between ANA and targets cells (i.e., membrane interaction and cell penetration) must be explored further to establish pathways specifically involved.

### 7.4 Induction of Disease by Antibodies and Animal Model of Immune Transfer

To date, there is no model of passive transfer of ANA in SSc, whether human to animal or animal to animal. These models may confirm the pathogenicity of ANA in the development or perennation of SSc.

### 7.5 Significance of the Pathogenicity of ANA in SSc

SSc is one of the most complex and heterogeneous connective tissue disease, whose pathophysiology includes genetic background, endogenous and exogenous factors, microvascular dysfunction, and innate and adaptative auto-immunity leading to fibrosis and various clinical phenotypes (145). ANA could be deleterious by initiating disease or by perpetuating an established process. ANA pathogenicity could also be influenced by the other hallmarks of SSc. For examples, VDJ mutation on B cells producing Aab may promote pathogenic Aab production (146). In SSc, genes susceptibility associated with distinct ANA subtypes is described (147). Moreover, hormonal environment is known to influence Aab, which may lead to tissue injury in SLE (148). The most convincing hypothesis is that ANA are pathogenic in SSc in certain patients, especially in ATA-positive patients. ANA may contribute to the perpetuation of the phenotypic trait observed in the patients, and their deleterious effect is probably dependent on other etiological factors of the disease.

## 8 Conclusion

ANA are strong diagnosis and prognosis biomarkers in SSc, but their pathogenicity is not yet established. One might think that the nuclear nature of their targets prevents any accessibility to autoantibodies. However, a new paradigm in whom ANA could be pathogenic in SSc emerges. Different potential mechanisms of pathogenicity have been described, but many aspects remain to be elucidated and deserve further research.

## Author Contributions

AC, LB, VK, DL, SD, and VS contributed to the conception and bibliography of the review. AC wrote the first draft of the manuscript and contributed to figures creation. LB created the figures. DL, SD, and VS made major revisions to the manuscript. All authors contributed to the article and approved the submitted version.

## Conflict of Interest

DL reports grants from GSK, Actelion, Boehringer Ingelheim, Takeda, CSL Behring, and Biocryst, outside the submitted work. VS reports consultancies and speaking fees from Boehringer Ingelheim and Grifols (less than $10 000) and research support from Actelion, Grifols, GSK, Octapharma, Pfizer, and Shire, outside the submitted work.

The remaining authors declare that the research was conducted in the absence of any commercial or financial relationships that could be construed as a potential conflict of interest.

## Publisher’s Note

All claims expressed in this article are solely those of the authors and do not necessarily represent those of their affiliated organizations, or those of the publisher, the editors and the reviewers. Any product that may be evaluated in this article, or claim that may be made by its manufacturer, is not guaranteed or endorsed by the publisher.

## Abbreviations

Aab, autoantibodies; ACA, anti-centromere antibodies; ADCC, autoantibody-dependent cell-mediated cytotoxicity; ANA, antinuclear antibodies; AFA, anti-fibroblast antibodies; anti-PDGFR, anti-platelet-derived growth factor receptor antibodies; anti-RNAP, anti-RNA polymerase III antibodies; ATA, anti-topoisomerase-I antibodies; dcSSc, diffuse cutaneous systemic sclerosis; ECM, extracellular matrix; FB, fibroblasts; FcγR, Fc gamma receptor; IVIg, intravenous immunoglobulins; lcSSc, limited cutaneous systemic sclerosis; PTM, post-translational modifications; SAID, systemic autoimmune disease; SLE, systemic lupus erythematosus; RTX, rituximab; SSc, systemic sclerosis; TLR, toll-like receptor; TOPO-I, topoisomerase-I; VEDOSS: very early diagnosis of SSc.
